# Recognition of tRNA^His^ in an RNase P-Free Nanoarchaeum

**DOI:** 10.1128/spectrum.04621-22

**Published:** 2023-02-22

**Authors:** Indira Rizqita Ivanesthi, Gita Riswana Nawung Rida, Aditya Aryandi Setiawibawa, Yi-Kuan Tseng, Arief Muammar, Chien-Chia Wang

**Affiliations:** a Department of Life Sciences, National Central University, Jungli District, Taoyuan, Taiwan; b Graduate Institute of Statistics, National Central University, Jungli District, Taoyuan, Taiwan; c Faculty of Biology, Universitas Gadjah Mada, Yogyakarta, Indonesia; Northwestern University

**Keywords:** aminoacyl-tRNA synthetase, protein synthesis, RNase P, thermophilic archaeum, tRNA

## Abstract

The 5′ extra guanosine with 5′-monophosphate at position -1 (G-1) of tRNA^His^ (p-tRNA^His^) is a nearly universal feature that establishes tRNA^His^ identity. G-1 is either genome encoded and retained after processing by RNase P (RNase P) or posttranscriptionally incorporated by tRNA^His^ guanylyltransferase (Thg1) after RNase P cleavage. However, RNase P is not found in the hyperthermophilic archaeum Nanoarchaeum equitans; instead, all of its tRNAs, including tRNA^His^, are transcribed as leaderless tRNAs with 5′-triphosphate (ppp-tRNAs). How N. equitans histidyl-tRNA synthetase (NeHisRS) recognizes its cognate tRNA (NetRNA^His^) is of particular interest. In this paper, we show that G-1 serves as the major identity element of NetRNA^His^, with its anticodon performing a similar role, though to a lesser extent. Moreover, NeHisRS distinctly preferred p-tRNA^His^ over ppp-tRNA^His^ (~5-fold difference). Unlike other prokaryotic HisRSs, which strongly prefer tRNA^His^ with C73, this enzyme could charge tRNAs^His^ with A73 and C73 with nearly equal efficiency. As a result, mutation at the C73-recognition amino acid residue Q112 had only a minor effect (<2-fold reduction). This study suggests that NeHisRS has evolved to disregard C73, but it still maintains its evolutionarily preserved preference toward tRNA^His^ with 5′-monophosphate.

**IMPORTANCE** Mature tRNA^His^ has, at its 5′-terminus, an extra guanosine with 5′-monophosphate, designated G-1. G-1 is the major recognition element for histidyl-tRNA synthetase (HisRS), regardless of whether it is of eukaryotic or prokaryotic origin. However, in the hyperthermophilic archaeum Nanoarchaeum equitans, all its tRNAs, including tRNA^His^, are transcribed as leaderless tRNAs with 5′-triphosphate. This piqued our curiosity about whether N. equitans histidyl-tRNA synthetase (NeHisRS) prefers tRNA^His^ with 5′-triphosphate. We show herein that G-1 is still the major recognition element for NeHisRS. However, unlike other prokaryotic HisRSs, which strongly prefer tRNA^His^ with C73, this enzyme shows almost the same preference for C73 and A73. Most intriguingly, NeHisRS still prefers 5′-monophosphate over 5′-triphosphate. It thus appears that the preference of HisRS for tRNA^His^ with 5′-monophosphate emerged very early in evolution.

## INTRODUCTION

Aminoacyl-tRNA synthetases (aaRSs) belong to a group of essential translation enzymes that are responsible for attaching amino acids to their cognate tRNAs. The resulting aminoacyl-tRNAs are delivered to ribosomes for genetic code deciphering via base pairing between the anticodon of tRNA and the codon of mRNA (1). Hence, the fidelity of protein translation is in part determined by the accuracy of aaRS-catalyzed tRNA aminoacylation. How aaRSs can correctly and specifically recognize their cognate tRNAs is critical for protein synthesis. AaRSs establish tRNA’s identity through a set of identity elements or determinants present in the tRNA. These identity elements could be single nucleotides, base pairs, or posttranscriptional modifications (2–4), which most often reside in the acceptor stem or anticodon loop of tRNA. For example, alanine tRNA (tRNA^Ala^) carries a primary identity element, G3-U70, in the acceptor stem (5), while histidine tRNA (tRNA^His^) carries a unique identity element, an extra G at position −1 (6).

tRNAs are transcribed by RNA polymerase as precursor molecules with extra sequences at their 5′ and 3′ termini. Precursor tRNAs must be properly processed before they can fulfill their function as adaptor molecules. Eukaryotic tRNA maturation involves a series of RNase-catalyzed steps, including the removal of 5′-leader and 3′-trailer sequences, the splicing of introns, the addition of a 3′-CCA end, and several specific base modifications (7). These modifications play an important role in stabilizing the tertiary structure of tRNAs (8). In addition to these universal modifications, a unique modification, 3′ to 5′ addition of an extra guanosine to position −1, occurs in most eukaryotic tRNA^His^ species (9).

**FIG 1:**
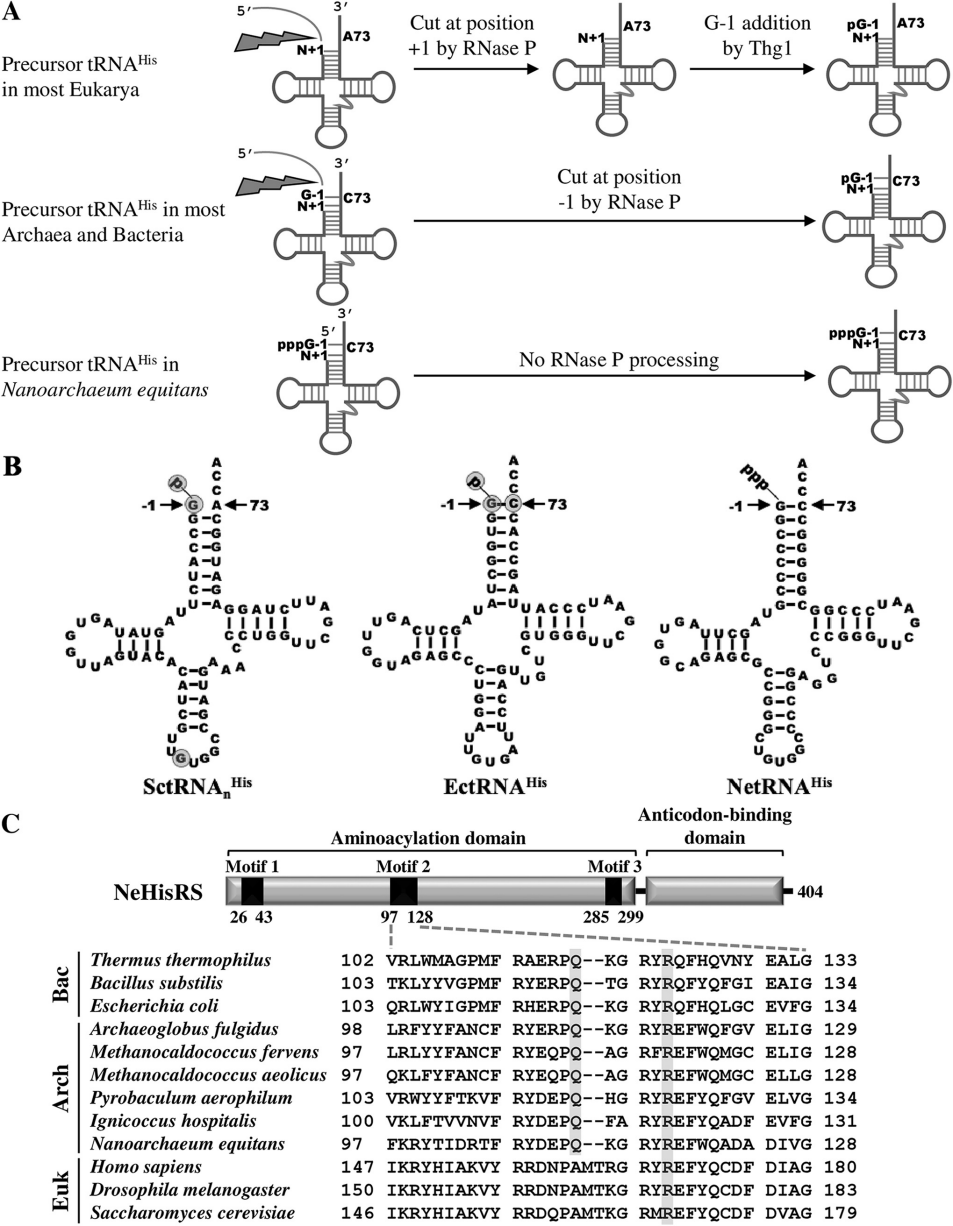
Commonalities and differences between NeHisRS and NetRNA^His^ and homologous molecules in other organisms. (A) Maturation of tRNA^His^ through different pathways. G-1 of tRNA^His^ is either genome encoded (in most archaea and bacteria) or acquired by posttranscriptional addition by Thg1 (in most eukarya). There is no RNase P processing in N. equitans, resulting in tRNA^His^ with 5′-triphosphorylated G-1. (B) Cloverleaf structures of various tRNAs^His^. Identity elements in SctRNA_n_^His^ and EctRNA^His^ are shaded. (C) Sequence alignment of motif 2 of HisRS. Amino acid residues that are involved in G-1-N73 recognition are shaded.

A previous study showed that G-1 is a primary and unique identity element of tRNA^His^ in nearly all organisms (6). In prokaryotes, G-1 is positioned across from C73, forming a Watson-Crick G-C base pair, while in eukaryotes, G-1 is positioned across from A73, forming a G-A mismatch (9). In archaea and bacteria, G-1 is most often genome encoded and retained after processing by RNase P, whereas in eukarya, G-1 is usually added posttranscriptionally by tRNA^His^ guanylyltransferase (Thg1) after RNase P cleavage (10) (Fig. 1A). Both pathways yield a mature tRNA^His^ species with 5′-monophosphorylated G-1 (p-tRNA^His^) (11). Previous studies have shown that the 5′-monophosphate is more important than the guanosine itself for recognition by HisRSs. Evidence shows that p-tRNA^His^ is ~14-fold more efficient than 5′-triphosphate tRNA^His^ (ppp-tRNA^His^) in histidylation by E. coli or yeast HisRS (12, 13). In addition to G-1, C73 is a strong identity element for tRNA^His^ recognition by E. coli HisRS (14).

Nanoarchaeum equitans is a thermophilic archaeum that grows best between 70°C and 98°C. This nanoarcheaum carries the smallest genome in the archaea domain (~490 kb) and is an obligate symbiont. It is unable to self-sustain but can grow in a coculture with the crenarchaeon Ignicoccus hospitalis (15). The small genome size of N. equitans appears to be an outcome of genome reduction. Analysis of the whole-genome sequence shows no gene encoding RNase P or Thg1 in this nanoarchaeum. In response to this genetic loss, each tRNA gene of N. equitans is preceded by a promoter, which is positioned 26 or 27 nucleotides upstream of the tRNA sequence. Transcription starts at −1 of tRNA^His^, tRNA^Tyr^, and tRNA^iMet^ and +1 of all other tRNAs. This strict promoter placement gives rise to leaderless mature tRNAs. In contrast to RNase P-processed tRNAs, which carry 5′-monophosphate termini, all nanoarchaeal tRNAs carry 5′-triphosphate termini (16). As 5′-monophosphate on G-1 usually performs a key role in tRNA^His^ recognition by HisRS, this finding prompted us to ask whether NeHisRS has developed a novel preference toward a 5′-triphosphate and how NeHisRS may actually recognize its cognate tRNA. Our results showed that NeHisRS has evolved to tolerate ppp-tRNA^His^, but it still maintains its evolutionarily preserved preference toward p-tRNA^His^.

## RESULTS

### NetRNA^His^ carries a 5′-triphosphorylated G-1.

Unlike most other organisms, N. equitans lacks RNase P, and therefore, its tRNA^His^ maturation depends entirely on strict promoter placement. This rigid arrangement allows production of leaderless and trailerless tRNA transcripts. As a result, the nascent N. equitans tRNA^His^ (NetRNA^His^) transcript contains a 5′-triphosphorylated G-1 and a 3′-CCA end (Fig. 1A and B). Previous studies suggested that G-1 and the discriminator base C73 in the acceptor stem may serve as the primary identity elements of bacterial and archaeal tRNAs^His^ (17), while G-1 and G34 (the first position of the anticodon) may serve as the primary identity elements of yeast tRNAs^His^ (13). G-1 and C73 are, respectively, recognized by an R and a Q residue (as R123 and Q118 in EcHisRS) located in the motif 2 loop of prokaryotic HisRSs. Because eukaryotic tRNAs^His^ contain A73 instead of C73, the Q residue is not conserved and is replaced by the tripeptide Ala-Met-Thr (AMT), or a related sequence, in eukaryotic HisRSs (17) (Fig. 1C).

### NeHisRS is a thermophilic enzyme that can efficiently charge tRNA^His^ with A73.

**FIG 2:**
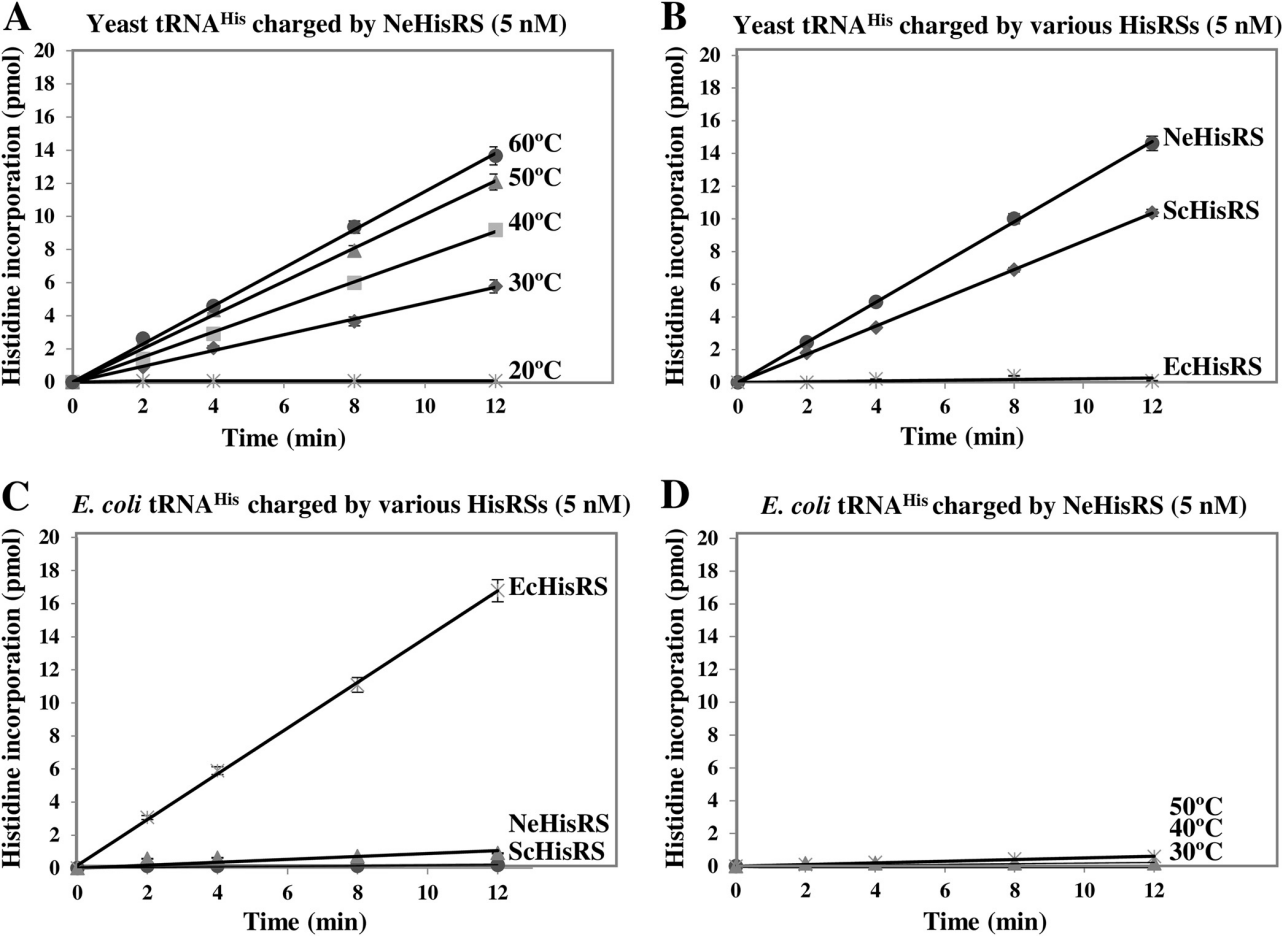
Aminoacylation assay using E. coli and yeast tRNAs. (A) Aminoacylation of unfractionated yeast tRNA by NeHisRS at various temperatures. (B) Aminoacylation of yeast unfractionated tRNA by various HisRSs. (C) Aminoacylation of unfractionated E. coli tRNA by various HisRSs. (D) Aminoacylation of unfractionated E. coli tRNA by NeHisRS at various temperatures. The data were obtained from three independent experiments and averaged. The error bars represent the SEM of triplicate experiments.

To explore whether the discriminator base N73 actually plays an important role in the recognition of tRNA^His^ by NeHisRS, *in vitro* aminoacylation assays were first carried out using unfractionated tRNA from yeast and Escherichia coli as the substrates. Yeast and E. coli tRNA^His^ isoacceptors possess A73 and C73, respectively (Fig. 1B). To find a suitable temperature for aminoacylation by NeHisRS, we first tried aminoacylation between 20°C and 60°C. As shown in Fig. 2A, the aminoacylation activity of NeHisRS gradually increased as the temperature rose from 20°C to 60°C. The aminoacylation activity at 50°C or 60°C was 2- to 3-fold higher than that at 30°C, while almost no activity was detected at 20°C. This confirmed NeHisRS as a thermophilic enzyme. Unless otherwise indicated, the aminoacylation assays were carried out at an ambient temperature (~25°C) for E. coli and yeast HisRSs and at 50°C for NeHisRS. Figure 2B shows that yeast HisRS efficiently charged yeast tRNA^His^ provided in a total tRNA fraction from yeast, whereas E. coli HisRS remained essentially inactive in this setup, implying the presence of a structural element in yeast tRNA^His^ that serves as an antideterminant for EcHisRS. From this perspective, it was surprising to find that the nanoarchaeal enzyme NeHisRS can efficiently charge yeast tRNA^His^ (with A73) despite the fact that its cognate tRNA possesses C73 (Fig. 2A and B).

As expected, EcHisRS could efficiently charge E. coli tRNA^His^. However, contrary to our anticipation, neither ScHisRS nor NeHisRS could charge E. coli tRNA^His^ to an appreciable level (Fig. 2C). Because the aminoacylation assays for NeHisRS were carried out at 50°C, we were worried that E. coli tRNA^His^ might be partially unfolded and become a poor substrate for aminoacylation at this temperature. To ease this concern, we next carried out the assay at a lower temperature. The results showed that no significant charging was observed between 20°C and 50°C for NeHisRS (Fig. 2D). This observation may be explained by the possible presence of an antideterminant in E. coli tRNA^His^, and future studies may explore this phenomenon in greater detail.

### NeHisRS prefers p-tRNA^His^ over ppp-tRNA^His^.

**FIG 3:**
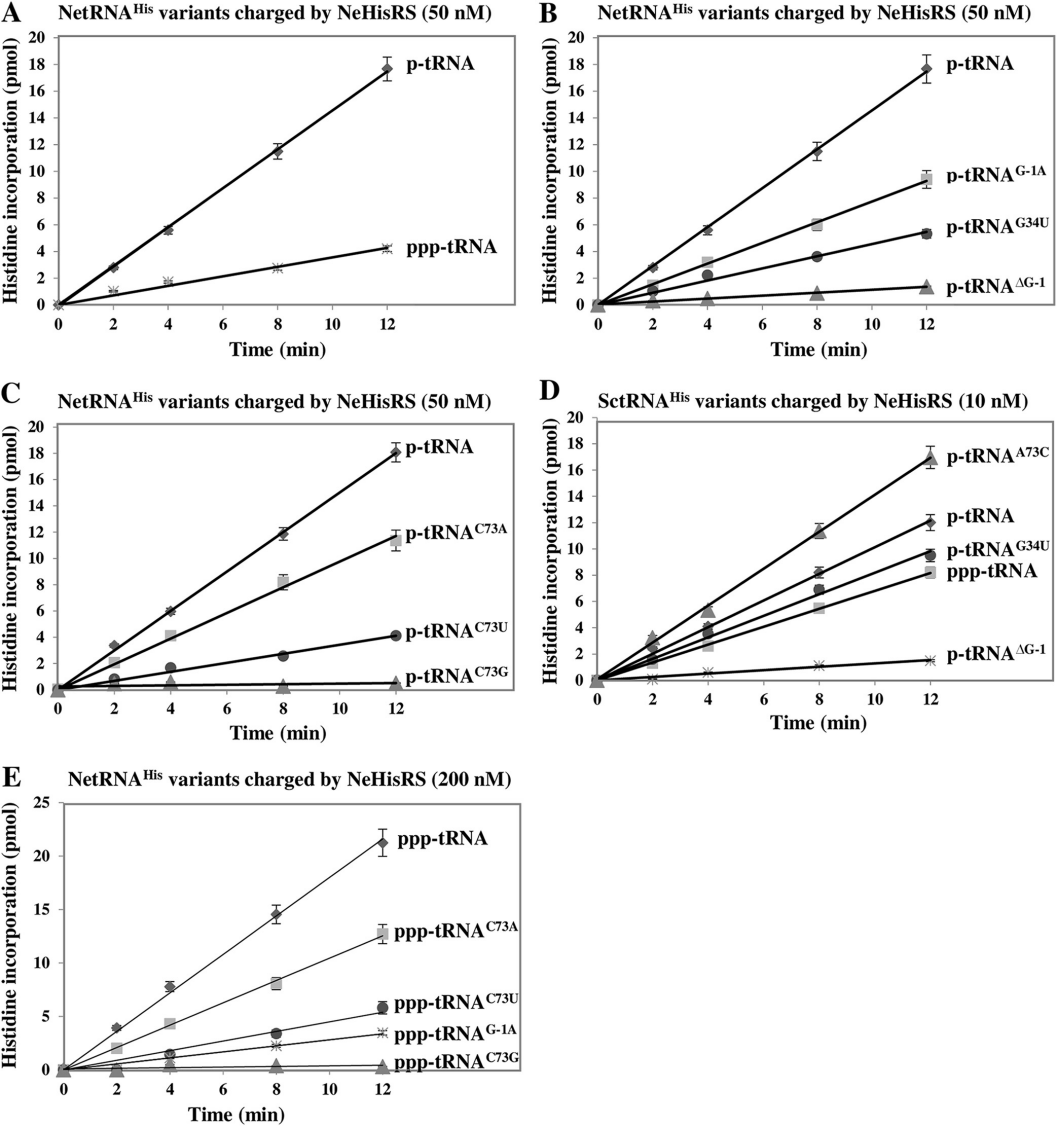
Aminoacylation assay using N. equitans tRNA^His^. (A) Aminoacylation of ppp-NetRNA^His^ and p-NetRNA^His^ by NeHisRS. (B) Aminoacylation of p-NetRNA^His^ mutants by NeHisRS. (C) Aminoacylation of C73 mutants of p-NetRNA^His^ by NeHisRS. (D) Aminoacylation of WT and mutant SctRNA_n_^His^ transcripts by NeHisRS. (E) Aminoacylation of G-1 and C73 mutants of ppp-NetRNA^His^ by NeHisRS. The data were obtained from three independent experiments and averaged. The error bars represent the SEM of triplicate experiments.

Despite the fact that the 5′-monophosphate of tRNA^His^ is the major determinant for E. coli and yeast HisRSs (13, 14), a considerable fraction of NetRNA^His^ carries a 5′-triphosphate (16). To test whether NeHisRS prefers its cognate tRNA with 5′-triphosphate, *in vitro*-transcribed NetRNAs^His^ with 5′-monophosphate and 5′-triphosphate were prepared and used as the substrates for aminoacylation. As shown in Fig. 3A, NeHisRS charged p-tRNA^His^ with efficiency ~5-fold higher than that for ppp-tRNA^His^. Thus, NeHisRS prefers tRNA^His^ with a 5′-monophosphate despite the presence of 5′-triphosphorylated cognate tRNA in N. equitans. This might be an inevitable consequence of the genetic loss of RNase P in this nanoarchaeum.

We next examined all potentially important identity elements, including G-1, G34 (the most influential position in the anticodon) (13), and C73 (the discriminator base), for their effect on aminoacylation by NeHisRS. As expected, deletion of G-1 from p-NetRNA^His^ drastically reduced its aminoacylation rate (~24-fold). However, mutation of G-1 to A had only a minor effect on its aminoacylation rate (~2-fold reduction) (Fig. 3B). Thus, it is the 5′-monophosphate on G-1, but not the guanosine itself, that is crucial for recognition by NeHisRS. In contrast to G-1, the anticodon is less crucial for recognition. Mutation of G34 to U reduced its aminoacylation rate ~4-fold (Fig. 3B). As for N73, mutation of C73 to A only reduced its aminoacylation rate ~1.5-fold, while mutation of C73 to U and G reduced its aminoacylation rate ~5- and ~40-fold, respectively (Fig. 3C). Taken together, these data suggest that G-1 and, to a much lesser extent, G34 are the major identity elements of NetRNA^His^. Similar scenarios were observed when these mutations were introduced into cytoplasmic SctRNA^His^. Interestingly, the yeast A73C variant performed even better than the parental p-tRNA (Fig. 3D). To evaluate whether certain base identities are more disadvantageous for aminoacylation of ppp-NetRNA^His^ relative to p-NetRNA^His^, we next carried out aminoacylation using ppp-NetRNA^His^ variants as the substrates. As shown in Fig. 3E, the C73 mutation to A, U, and G in ppp-NetRNA^His^ reduced aminoacylation 2-, 4-, and 40-fold, respectively, a pattern similar to that observed for C73 mutation in p-NetRNA^His^. Despite the fact that the G-1-A mutation in p-NetRNA^His^ reduced aminoacylation only 2-fold, a similar mutation in ppp-NetRNA^His^ reduced aminoacylation up to 7-fold.

To obtain the Michaelis constant (*K_m_*) and turnover number (*k*_cat_) values for aminoacylation of wild-type (WT) and mutant NetRNAs^His^ by NeHisRS, we performed a kinetic assay under conditions as described in Materials and Methods. As shown in Table 1, aminoacylation of p-tRNA^His^ was ~5-fold more efficient (*k*_cat_/*K_m_*) than that of ppp-tRNA^His^, and the difference in catalytic efficiency was mainly attributed to *K_m_* discrimination. That is, NeHisRS displayed a lower *K_m_* value (~3-fold) for p-tRNA^His^ than for ppp-tRNA^His^. In addition, mutation of C73 to A in p-tRNA^His^ caused only a 1.7-fold decrease in aminoacylation efficiency (*k*_cat_/*K_m_*). This suggests that C73 in NetRNA^His^ does not play as critical a role as previously anticipated in prokaryotic tRNA^His^. Consistent with the time course assay (Fig. 3), mutation of G34 to U reduced aminoacylation efficiency ~5-fold, but deletion of G-1 reduced aminoacylation efficiency up to 88-fold (Table 1).

**TABLE 1:** Kinetic parameters for aminoacylation of NetRNA^His^ by NeHisRS and Q112A

NetRNA^His^	*K*_cat_ (s^−1^)	*K_m_* (μM)	*K*_cat_/*K_m_* (μM^−1^s^−1^)	Relative efficiency (fold)
NeHisRS				
ppp-tRNA	0.32 ± 0.05	10.81 ± 1.16	0.03	1.0
p-tRNA	0.52 ± 0.08	3.50 ± 0.21	0.15	5.0
p-tRNA^C73A^	0.34 ± 0.06	3.71 ± 0.42	0.09	3.0
p-tRNA^ΔG-1^	0.002 ± 0.0005	1.21 ± 0.10	0.0017	0.057
p-tRNA^G34U^	0.11 ± 0.02	3.52 ± 0.22	0.03	1.0
Q112A				
p-tRNA	0.22 ± 0.02	1.92 ± 0.21	0.11	3.7
p-tRNA^C73A^	0.14 ± 0.02	2.21 ± 0.23	0.06	2.0

### The conserved Q residue in the motif 2 loop of NeHisRS is dispensable for tRNA^His^ recognition.

**FIG 4:**
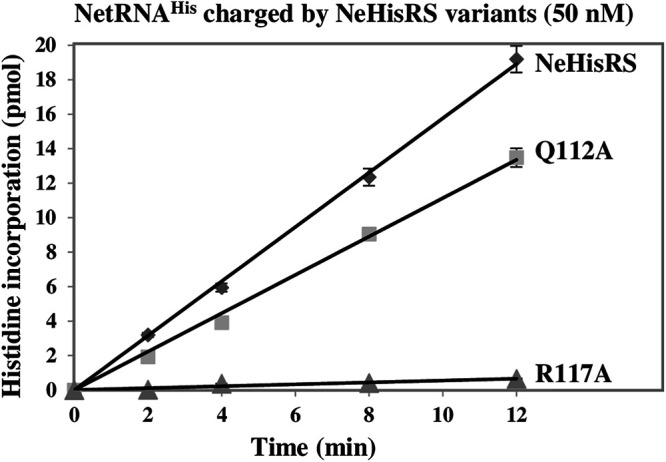
Aminoacylation assay for Q112A and R117A. Mutations were individually introduced into NeHisRS, and the resultant mutants’ aminoacylation activities were analyzed using WT p-NetRNA^His^ as the substrate. The data were obtained from three independent experiments and averaged. The error bars represent the SEM of triplicate experiments.

Previous studies showed that Q118 (part of a tRNA-binding pocket) of E. coli HisRS plays a key role in the recognition of C73 (the discriminator base) (17, 18). Despite the fact that NeHisRS also contains the corresponding Q residue (as Q112) in its motif 2 loop, our study showed that mutation of C73 to A in NetRNA^His^ had a very mild effect (<2-fold reduction) on aminoacylation by this enzyme (Fig. 3C and Table 1). To gain more insight, we mutated the corresponding amino acid residue Q112 in the motif 2 loop of NeHisRS (Fig. 1C) and analyzed the mutant’s aminoacylation activity. Mutation of Q112 to A resulted in a <2-fold decrease in aminoacylation of p-NetRNA^His^ with C73 (Table 1 and Fig. 4). Moreover, this mutant could charge p-NetRNA^His^ with A73 with a similar efficiency (only ~2-fold reduction) (Table 1). These results suggest that Q112 in NeHisRS does not play a similarly important role in tRNA^His^ recognition as Q118 in EcHisRS. This feature is of particular interest for a nondual function enzyme.

We next mutated the G-1-recognition amino acid residue R117 of NeHisRS to assess its role in tRNA^His^ recognition. As shown in Fig. 4, mutation of R117 to A led to an almost inactive enzyme (~40-fold reduction in aminoacylation) (Fig. 4), further attesting to the leading role of G-1 in NetRNA^His^ recognition.

### NeHisRS efficiently rescues the mitochondrial defect of a yeast HisRS KO strain.

Since NeHisRS showed only slight preference toward C73 over A73 (Fig. 3 and Table 1), we wondered whether this enzyme can functionally substitute for yeast cytoplasmic and mitochondrial HisRS activities *in vivo*. To this end, the gene encoding NeHisRS was codon optimized and cloned into a high-copy-number yeast expression vector, and the resultant plasmid clones were transformed into a yeast *HTS1* knockout (KO) strain for complementation assays at 30°C. We used yeast and E. coli HisRSs as positive controls in the functional assay.

**FIG 5:**
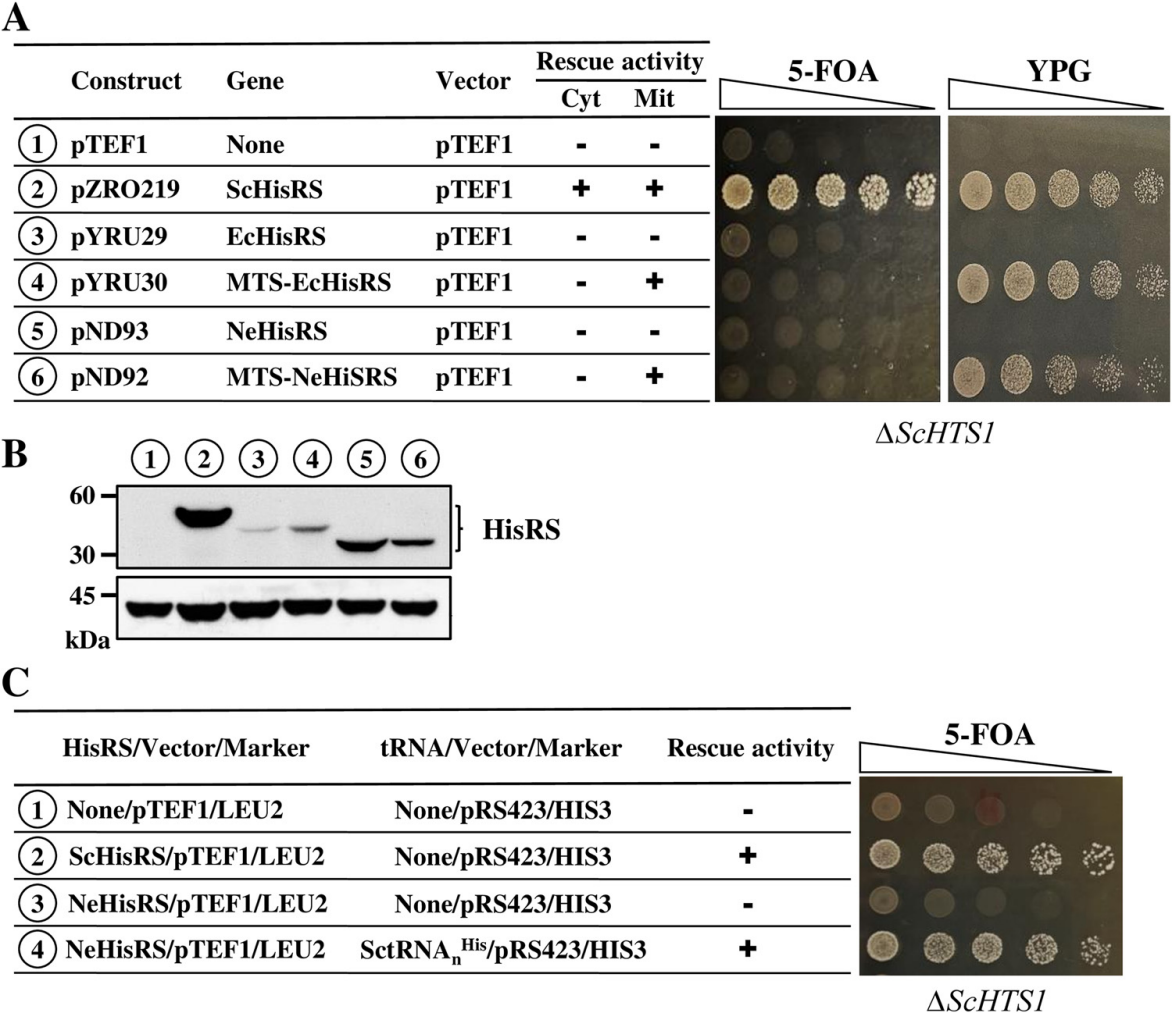
Rescue of the genetic loss of yeast *HTS1*. (A) Summary of the HisRS constructs and their rescue activities. The symbols “+” and “−”, respectively, denote positive and negative complementation. Growth on 5-FOA and YPG, respectively, indicates complementation of the cytoplasmic and mitochondrial HisRS activities. (B) Western blotting. Expression of HisRS from plasmids was probed with an HRP-conjugated anti-His_6_ tag antibody. Numbers 1 to 6 (circled) shown represent constructs shown in panel A. (C) Co-overexpression of NeHisRS and SctRNA_n_^His^. pTEF1 is a high-copy-number yeast vector with a *LEU2* marker, while pRS423 is a high-copy-number yeast vector with a *HIS3* maker.

As shown in Fig. 5A, ScHisRS successfully rescued the growth defects of the KO strain on both 5-fluoroorotic acid (5-FOA) and yeast extract-peptone-glycerol (YPG). In contrast, EcHisRS was unable to rescue the growth defect of the KO strain on 5-FOA and YPG, but fusion of a heterologous mitochondrial-targeting signal (MTS) enabled the enzyme (MTS-EcHisRS) to be imported into mitochondria and restore the growth phenotype of the KO strain on YPG (17). To our surprise, relatively low expression of MTS-NeHisRS could effectively support the growth of the null allele strain on YPG (Fig. 5A and B). In contrast, NeHisRS failed to confer a positive growth phenotype on 5-FOA despite relatively high expression. We hypothesized that this negative phenotype on 5-FOA is caused by the limited aminoacylation efficiency of the heterologous NeHisRS enzyme combined with a higher histidylation demand of protein synthesis in the cytoplasm. Investigating further, we cotransformed a second test plasmid that overexpresses yeast cytoplasmic tRNA^His^ (SctRNA_n_^His^) into the KO strain and tested the growth phenotype of the resultant cotransformants on 5-FOA. As shown in Fig. 5C, co-overexpression of NeHisRS and SctRNA_n_^His^ (with A73) successfully restored the growth phenotype of the KO strain on 5-FOA, further emphasizing the ability of NeHisRS to charge tRNA^His^ with A73. It is worth mentioning that NeHisRS has relatively low activity at the temperature (30°C) used for complementation (Fig. 2A).

## DISCUSSION

### NeHisRS prefers tRNA^His^ with 5′-monophosphate.

In E. coli, a 5′-triphosphate is known to reduce aminoacylation of tRNA^His^ (~14-fold) (12), and complete 5′-dephosphorylation of tRNA^His^ to 5′-OH-tRNA^His^ resulted in an ~510-fold decrease in aminoacylation (18), demonstrating the functional importance of the 5′-monophosphate. Despite the fact that a considerable fraction of mature NetRNA^His^ carries a 5′-triphosphate (16), NeHisRS still prefers p-NetRNA^His^ over ppp-NetRNA^His^, but with a moderate 5-fold difference (Table 1). Thus, it appears that coevolution of NeHisRS and NetRNA^His^ has rendered the enzyme more flexible to the 5 terminus of tRNA^His^, as the N. equitans system is somewhat more tolerant (~3-fold) toward 5′-triphosphate than the E. coli system. In addition to tRNA^His^, two more tRNA species in this nanoarcheaum are known to possess a purine nucleotide at position −1, tRNA^Tyr^ and tRNA^iMet^. However, A-1 in tRNA^iMet^ and G-1 in tRNA^Tyr^ appear to not play a major role in aminoacylation (15, 16). The cocrystal structure of Thermus thermophilus HisRS in complex with tRNA^His^ containing a 5′-triphosphate revealed that the G-1 α-phosphate interacts with R122 and R7, the β-phosphate with R122, and the γ-phosphate with R7 and R74 (19). Among these interacting amino acid residues, R122 and R7 are conserved in NeHisRS (as R117 and R6, respectively), while R74 diverged to K72 in NeHisRS, although it preserved the feature of a positively charged side chain.

### C73 is dispensable for recognition of NetRNA^His^.

In E. coli, C73 is a strong identity element for tRNA^His^ recognition. Mutation of C73 to A leads to a ~96-fold reduction in aminoacylation (14). Likewise, mutation of the C73-recognition amino acid residue Q118 to E reduces aminoacylation ~7-fold toward WT tRNA^His^ (18, 20). In contrast, yeast HisRS can efficiently charge both cytoplasmic (with A73) and mitochondrial (with C73) tRNA^His^ isoacceptors (21). Three amino acids (A, M, and T), instead of Q, are located at the presumed interaction site (Fig. 1). As a result, mutation of A73 to C in cytoplasmic tRNA^His^ or C73 to A in mitochondrial tRNA^His^ resulted in a <3-fold change in aminoacylation (17). Thus, N73 does not play a significant role in tRNA^His^ recognition in yeast. From this perspective, it is surprising that NeHisRS, a nondual function archaeal HisRS, also efficiently recognizes both C73 and A73, raising the possibility that the enzyme interacts with the 4- and 6-amino groups of C73 and A73, respectively.

### G-1 is the primary identity element of NetRNA^His^.

G-1 is known for being a key determinant of tRNA^His^ in nearly all organisms. Existence of G-1 could facilitate rejection of noncognate synthetases (18). Deletion of G-1 from S. cerevisiae and E. coli tRNAs^His^ drastically reduces aminoacylation efficiency (*k*_cat_/*K_m_*) ~740- and ~250-fold, respectively (6, 13). Likewise, deletion of G-1 from human tRNA^His^ leads to a defective tRNA unsuitable for aminoacylation (17). A similar effect was observed for NetRNA^His^, where deletion of G-1 from NetRNA^His^ reduced aminoacylation efficiency up to 88-fold (Table 1). Interestingly, substitution of this nucleotide with A in p-NetRNA^His^ reduced aminoacylation only ~2-fold (Fig. 3B), but a similar mutation in ppp-NetRNA^His^ reduced aminoacylation up to ~7-fold (Fig. 3E).

As for the anticodon, G34 (the most influential position in the anticodon) is considered more important than C73, but not as vital as G-1 in NetRNA^His^ recognition. Mutation of G34 to U reduced aminoacylation efficiency 5-fold (Table 1). In contrast, G34 plays a much more important role in the recognition of yeast cytoplasmic tRNA^His^. Mutation of G34 to U in SctRNA^His^ caused an ~75-fold reduction in aminoacylation (13). However, a similar mutation in EctRNA^His^ had little effect on aminoacylation (22). It is also interesting to mention that G34U mutation in NetRNA^His^ affects the *k*_cat_ instead of the *K_m_*, a scenario similar to that of C. elegans cytoplasmic HisRS (23). Perhaps this mutation alters the positioning of the bound tRNA and its CCA end, thereby affecting catalytic efficiency.

### Coevolution between NetRNA^His^ and NeHisRS.

N. equitans carries the smallest genome (~490 kb) with the highest coding density of any genome sequenced thus far (15). It is believed that this small genome is the outcome of genome reduction associated with the obligatory parasitic lifestyle of this symbiont. The universal conservation of RNase P implies that this enzyme is ancient and likely existed in the last universal common ancestor. It has thus been hypothesized that progenitors of N. equitans possessed this enzyme but lost it later during evolution (16). Meanwhile, the rapid evolutionary tempo of N. equitans enabled its tRNA promoters to be positioned in such a way that transcription always starts at position +1 (or position −1 in the cases of tRNA^Tyr^, tRNA^iMet^, and tRNA^His^) of mature tRNA transcripts. As a result, all contemporary nanoarchaeal tRNA transcripts are transcribed as leaderless tRNAs with 5′-triphosphate termini (16). To maintain normal protein synthesis, NeHisRS has adapted to its cognate tRNA^His^ with 5′-triphosphate. However, it still preserves its ancient preference toward 5′-monophosphorylated tRNA^His^.

## MATERIALS AND METHODS

### Construction of plasmids.

Cloning of the gene encoding NeHisRS into pTEF1 (a high-copy-number yeast shuttle vector with a strong *TEF1* promoter, a *LEU2* marker, and a 2μ replication origin) followed a standard protocol. In brief, the open reading frame encoding NeHisRS was retrieved from NCBI and submitted to a gene synthesis process by a manufacturer. The resultant DNA fragment was subcloned into the SpeI/XhoI sites of pTEF1. Fusion of a heterologous MTS to HisRS followed a strategy described earlier (24). A DNA sequence encoding the amino acid residues 1 to 46 of the mitochondrial precursor form of yeast valyl-tRNA synthetase was PCR amplified as an XbaI-SpeI fragment and then inserted into the 5′ SpeI site of the HisRS gene.

For protein purification, the gene encoding NeHisRS (or its mutant) was cloned into pET21b (an E. coli expression vector with a T7 promoter) following a similar protocol. The plasmid carrying the target gene was transformed into an E. coli expression strain, BL21-CodonPlus (DE3), and the bacterial culture was induced with isopropyl β-d-1-thiogalactopyranoside (1 mM). The His_6_-tagged protein was purified to homogeneity through Ni-nitrilotriacetic acid (NTA) column chromatography (23). Western blotting followed a standard protocol using a horseradish peroxidase (HRP)-conjugated anti-His_6_ tag antibody as a probe (25).

### Complementation assay for cytoplasmic activity on 5-FOA.

A haploid *HTS1* knockout strain (*MAT*α, *hts1*::*kanMX4*, *his3*Δ*1 leu2*Δ*0 lys2*Δ*0 ura3*Δ*0*) was constructed earlier (17). Yeast *HTS1* is a dual-function gene that encodes both the cytoplasmic and mitochondrial forms of HisRS (21). In addition, yeast cytoplasmic and mitochondrial tRNA^His^ isoacceptors contain A73 and C73, respectively. To test the cytoplasmic HisRS activity of a heterologous gene, a test plasmid carrying the target gene was transformed into the *HTS1* knockout strain (with a maintenance plasmid), and the resultant transformants were plated on a plate containing 5-FOA (1 mg/mL) (26). The maintenance plasmid carries a wild-type *HTS1* gene and a *URA3* marker. Starting from an optical density (*A*_600_) of 1.0, cell cultures were 3-fold serially diluted, and 10-μL aliquots of each dilution were spotted onto the 5-FOA plate. The plate was then incubated at 30°C for 3 days. Because 5-FOA can be converted by yeast to a toxic compound in the presence of *URA3* (carried by the maintenance plasmid), the transformants must first evict the maintenance plasmid in order to survive. Without the maintenance plasmid, the transformants could not grow on 5-FOA unless the test plasmid provided a functional cytoplasmic HisRS. The strategy used to determine the rescue activity of a nonorthogonal pair of HisRS/tRNA^His^ was previously described (23).

### Complementation assay for mitochondrial activity on YPG.

To test the mitochondrial HisRS activity of a heterologous gene, a test plasmid (carrying the target gene and an *LEU2* marker), the first maintenance plasmid described above (carrying a wild-type *HTS1* and a *URA3* marker), and a second maintenance plasmid (carrying a *HIS3* marker and an initiator mutant of *HTS1* that encodes only the cytoplasmic form of HisRS) were cotransformed into the yeast *HTS1* knockout strain. The first maintenance plasmid was evicted from the cotransformants in the presence of 5-FOA, while the second maintenance plasmid was retained. Following 5-FOA selection, the surviving cotransformants were further spotted on a yeast extract-peptone-glycerol (YPG) plate. The plate was incubated at 30°C for 3 days to see whether the cotransformants could grow. Because glycerol is a nonfermentable carbon source, yeast can only metabolize this sugar alcohol through oxidative phosphorylation. Thus, the cotransformants could not survive on a YPG plate unless the test plasmid provided a functional mitochondrial HisRS.

### Preparation of tRNA^His^ transcripts.

*In vitro* transcription of tRNA^His^ followed a previously described protocol (6). The transcription template was enriched by PCR amplification of the insert (containing a T7 promoter and the tRNA gene). The *in vitro* transcription reaction of tRNA^His^ (with a 5′ GTP) was carried out at 37°C for 3 h using 0.3 μM T7 RNA polymerase in a buffer containing 20 mM Tris-HCl (pH 8.0), 150 mM NaCl, 20 mM MgCl_2_, 5 mM dithiothreitol (DTT), 1 mM spermidine, and 2 mM each NTP. For preparation of tRNA^His^ with a 5′ GMP, an extra 20 mM GMP was added to the reaction. The tRNA^His^ transcript was purified using an 8-M urea-10% polyacrylamide gel electrophoresis. Following ethanol precipitation and vacuum drying, the tRNA pellet was dissolved in 1× TE buffer (20 mM Tris-HCl [pH 8.0] and 1 mM EDTA) and refolded by heating to 80°C and gradually cooled to room temperature after addition of 10 mM MgCl_2_. Approximately 80% of *in vitro*-transcribed tRNA^His^ was active in aminoacylation.

### Aminoacylation assay.

Aminoacylation reactions were carried out at 50°C for NeHisRS (unless otherwise indicated) or 25°C for E. coli and yeast HisRSs in a buffer containing 50 mM HEPES (pH 7.0); 50 mM KCl; 15 mM MgCl_2_; 3 mM dithiothreitol; 4 mM ATP; 5 μM *in vitro*-transcribed tRNA^His^, 100 μM E. coli, or yeast unfractionated tRNA; and 26.25 μM histidine (6.25 μM [^14^C]histidine; PerkinElmer, Waltham, MA, USA). tRNA^His^ accounts for ~3.2% and ~4.3% in yeast and E. coli unfractionated tRNAs, respectively. Aminoacylation assay followed a previously described protocol (17). The specific activity of [^14^C]histidine used was 325 mCi/mmol. The final concentrations of the enzymes used in the reactions are indicated on top of the figures. Reactions were quenched by spotting 10-μL aliquots of the reaction mixture onto Whatman filters (Maidstone, Kent, UK) that have been presoaked in 5% trichloroacetic acid (TCA) and 2 mM histidine. The filters were washed three times for 15 min each in ice-cold 5% TCA before liquid scintillation counting. Data were obtained from three independent experiments and averaged.

Kinetic parameters for the histidylation of tRNA were determined by measuring the initial rate of charging over the first 2 min. Initial rates of aminoacylation were determined at 50°C with NetRNA^His^ concentrations ranging from 1 to 30 μM and NeHisRS concentrations ranging from 10 to 1,000 nM. Each initial rate at a given substrate concentration was determined, and the slope of line was derived by linear regression. The parameters were derived from Lineweaver-Burk plots. Error values represent standard deviations. Data were obtained from three independent experiments and averaged (17, 27).
